# Using nomogram of the Barcelona Clinic Liver Cancer system for treatment selection in patients with stage C hepatocellular carcinoma

**DOI:** 10.1186/s12885-018-4202-3

**Published:** 2018-03-14

**Authors:** Chia-Yang Hsu, Po-Hong Liu, Shu-Yein Ho, Cheng-Yuan Hsia, Praneeth Kudaravalli, Yun-Hsuan Lee, Yi-You Chiou, Ya-Ju Tsai, Yi-Hsiang Huang, Teh-Ia Huo

**Affiliations:** 1 Department of Medicine, Taipei Veterans General Hospital, Taipei, Taiwan; 2 Department of Surgery, Taipei Veterans General Hospital, Taipei, Taiwan; 3 Departments of Radiology, Taipei Veterans General Hospital, Taipei, Taiwan; 4 Faculty of Medicine, National Yang-Ming University School of Medicine, Taipei, Taiwan; 5 Institute of Pharmacology, National Yang-Ming University School of Medicine, Taipei, Taiwan; 6 Institute of Clinical Medicine, National Yang-Ming University School of Medicine, Taipei, Taiwan; 7 Department of Internal Medicine, University of Nevada School of Medicine, Reno, NV USA; 8 Harvard T.H. Chan School of Public Health, Boston, MA USA; 9 Renown Regional Medical Center, Reno, NV USA; 10 Division of Gastroenterology and Hepatology, Department of Medicine, Taipei Veterans General Hospital, Taipei, Taiwan

**Keywords:** Barcelona clinic liver Cancer, Hepatocellular carcinoma, Nomogram, Staging system

## Abstract

**Background:**

The nomogram of the Barcelona Clinic Liver Cancer (BCLC) for hepatocellular carcinoma (HCC) has been used for outcome prediction. Patients with BCLC stage C HCC often undergo anti-cancer therapy against current treatment guidelines in real world practice. We aimed to use the nomogram to provide guidance on treatment selection for BCLC stage C patients.

**Methods:**

A total of 1317 patients with stage C HCC were retrospectively analyzed and divided into four groups by nomogram points. One-to-one matched pairs between patients receiving different treatments were generated by the propensity score with matching model within these groups. Survival analysis was performed by Kaplan-Meier method with log-rank test.

**Results:**

Patients with higher nomogram points were more often treated with targeted or supportive therapies (*p* <  0.001). Patients receiving targeted or supportive therapies had a decreased survival compared to patients undergoing aggressive treatments (surgical resection, ablation, transarterial chemo-embolization or transplantation) across all four groups (*p* <  0.001). After matching for baseline differences in the propensity model, patients receiving different treatments had comparable age, gender, etiology of liver disease, tumor burden, severity of cirrhosis and performance status. Survival analyses were re-performed and disclosed that patients with nomogram points < 15 had better overall outcome after aggressive treatments (*p* <  0.05). For patients with nomogram points > 15, there was no significant difference in survival between patients receiving two different treatment strategies.

**Conclusions:**

The nomogram of BCLC system is a feasible tool to help stage C HCC patients to select primary anti-cancer treatment in pursuance of better overall survival.

## Background

Hepatocellular carcinoma (HCC) is one of the most common cancers in the world. The Barcelona Clinic Liver Cancer (BCLC) staging system has been recommended to be the prognostic model and also the allocating tool of primary anti-cancer treatment by major academic societies for liver diseases [1, 2]. For patients with BCLC stage C HCC, targeted therapy (sorafenib) has been suggested as the treatment of choice after two large randomized studies were published [3, 4]. However, a substantial proportion of patients may select more aggressive anti-cancer treatments to prolong their survival [5, 6]. Alternatively, it is also conceivable that patients receiving aggressive therapies could have a higher risk of treatment-associated complications that may sometimes lead to a shortened survival [7]. Currently, there is no clear consensus to suggest patients with BCLC stage C to receive more aggressive treatments instead of targeted therapy.

Recently, the nomogram of the BCLC system, which is designed to provide individualized prediction of patient survival, has been proposed and externally validated [8–10]. By using well-known clinical parameters of the BCLC system (performance status [PS], tumor burden and severity of cirrhosis), there is no additional lab test or complicated mathematic equation required to use this tool. The nomogram can also further stratify HCC patients within the same BCLC stage into multiple levels to reflect different disease severity, and may be helpful to design an optimal treatment strategy in real-world practice. This study aims to specifically investigate if aggressive treatments (surgical resection, transplantation, ablation and transarterial chemoembolization [TACE]) can improve the long-term prognosis of patients with BCLC stage C HCC. Also, the nomogram of BCLC is applied to provide better prognostic stratification and to facilitate the selection of treatment for stage C HCC patients.

## Methods

### Patients

During a 14-year period between 2002 and 2016, 1317 newly diagnosed BCLC stage C HCC patients in our hospital were identified and retrospectively analyzed in this study. Etiology of underlying liver disease, serum biochemistry, number and size of tumor(s), PS, severity of liver cirrhosis and cancer stage were comprehensively recorded at the time of diagnosis. The survival status of all patients was checked every 2–3 months after enrollment and was confirmed by using the database of National Cancer Registry, Taiwan. This study has been approved by the institutional review board (IRB) of Taipei Veterans General Hospital. Before analysis, waiver of consent form from each patient was obtained as justified by the IRB, and patient information was blinded and de-identified.

### Diagnosis and definitions

Findings of typical radiological features in at least two imaging modalities including magnetic resonance imaging (MRI), contrast-enhanced dynamic computed tomography (CT), ultrasound and hepatic arterial angiography, or by a single positive imaging study associated with serum α-fetoprotein (AFP) level ≥ 400 ng/mL or histological confirmation were used to diagnose HCC [11]. Daily consumption of at least 40 g of alcohol for 5 years or more was considered alcoholic liver disease [12]. Patients who were seropositive for anti-hepatitis C virus (HCV) antibody were classified as HCV-related HCC. Hepatitis B virus (HBV)-related HCC was defined as seropositive for HBsAg. Vascular invasion was diagnosed by the presence of thrombus adjacent to the tumor in portal system by at least two imaging modalities. Total tumor volume was calculated based on tumor diameter of every HCC nodules [13]. The Eastern Cooperative Oncology Group (ECOG) criteria were used to evaluate the overall physical status of each patient [14]. Patients with single tumor smaller than 2 cm in size were coded as tumor burden grade 0. Patients with tumor burden beyond grade 0 and within the Milan criteria (one nodule < 5 cm, up to 3 nodules < 3 cm, no vascular invasion or extrahepatic involvement) were classified as tumor burden grade 1 [10, 15]. Patients were recorded as tumor burden grade 3 if lymph node involvement, vascular invasion, or distant metastasis were confirmed at the time of diagnosis. All remaining patients were coded as tumor burden grade 2 [10]. Chest CT scan was performed to detect metastatic lesion(s) and lymph node involvement. Bone metastasis of HCC was surveyed by bone scan and confirmed by MRI if indicated. Surgical resection, ablation, liver transplantation and TACE were collectively defined as aggressive treatments, and targeted therapy and supportive treatment were classified together as the reference group based on the original design of BCLC system. All clinical data were recorded at the time of diagnosis before specific anti-cancer therapy was performed.

### Treatment

General criteria of surgical resection were (1) patients with tumor involving no more than 3 Healey’s segment, (2) Child-Turcotte-Pugh (CTP) class A, and (3) no main portal vein trunk involvement or distant metastasis. Liver transplantation was considered in patients fulfilling the Milan criteria [15]. TACE was performed depending on the size and number of tumor nodules as previously reported [5, 7]. Seldinger’s technique of arterial embolization was performed as the standard TACE procedure. After tumor stain was identified, infusion of a mixture of 20–30 mg adriamycin (Carlo Erba, Milan, Italy) and 5–10 mL Lipiodol (Laboratoire Guerbet, Villepinte, France) was performed after the artery supplying the tumor was catheterized superselectively. Sufficient amount of emulsion and 2–3 mm strips of Gelfoam (Upjohn, Kalamazoo, MI) were delivered to the tumor area until complete flow stagnation was achieved.

### Statistics

Continuous demographic characteristics were compared with the Mann-Whitney ranked sum test. Categorical data were compared with the chi-squared or Fisher exact tests. The comparison of survival distributions was performed by using the Kaplan-Meier method with a log-rank test. A two-tailed *p* value less than 0.05 was considered statistically significant.

The propensity score was generated by using a logistic regression, which calculated the possibility of each patient to receive aggressive or targeted/supportive treatments. Possible variables associated with long-term survival and treatments allocation, including age, gender, severity of cirrhosis, PS, vascular invasion, tumor burden, renal function, serum AFP level, diabetes mellitus were included comprehensively for propensity score generation. To compare the association between experimental variable (anti-cancer treatment) and response (survival), one-to-one pairs were selected by using the propensity score and greedy algorithm to reduce potential biases in subsequent survival analysis [16, 17]. The greedy algorithm used in this study performed 5 to 1 digit matching mechanism; *p*-value and standardized difference were used to evaluate the goodness of this propensity score with one-to-one matching model. All statistical analyses were conducted with the SAS 9.4 (SAS Institute Inc., Cary, NC, USA).

## Results

### Patient characteristics

The baseline patient characteristics are provided in Table 1. Their mean age was 64 years, and 78% of them were male. Hepatitis B (55%) was the most common etiology of chronic liver disease. There were 41% patients who had multiple tumors, and 64% of patients had a main tumor diameter of more than 5 cm. Sixty-nine percent of patients were classified as CTP class A, and 24%, 53% and 23% of patients had PS 0, 1 and 2, respectively. Vascular invasion was documented in 45% of patients, and 27% of patients were diabetic. A total of 15% of patients were confirmed with distant metastasis at study enrollment.

**Table 1:** Baseline demographics of study patients

Number of patients	1317
Age (years, mean ± standard deviation [SD])	64 ± 13
Male/female (%)	78/22
Etiology of cirrhosis (%)	
Hepatitis B	721 (55)
Hepatitis C	349 (27)
Alcoholism	308 (23)
Serum biochemistry (mean ± SD)	
Albumin (g/dL)	3.6 ± 0.6
Bilirubin (mg/dL)	1.4 ± 2.2
Creatinine (mg/dL)	1.2 ± 1.3
Estimated glomerular filtration rate (ml/min/1.73m^2^)	78 ± 36
International normalized ratio of prothrombin time	1.1 ± 0.1
Child-Turcotte-Pugh class A/B (%)	69/31
Number and size of tumor (%)	
Single/multiple	59/41
≤ 5 cm/ > 5 cm	36/64
Total tumor volume (cm^3^, mean ± SD [median])	566 ± 861 (193)
Vascular invasion (%)	591 (45)
Metastasis/lymph node	198 (15)
α-fetoprotein (ng/mL, mean ± SD [median])	34,136 ± 177,031 (118)
Tumor burden 0/1/2/3 (%)	4.5/17.2/28.3/50
Ascites (%)	398 (30)
Performance status 0/1/2 (%)	24/53/23
Diabetes mellitus (%)	358 (27)
Treatment modality (%)	
Resection	282 (21)
Transplantation	4 (0.3)
Ablation	149 (11)
Transarterial chemo-embolization	437 (33)
Targeted therapy	107 (8)
Best supportive care	338 (26)

Of all patients, 21% of patients received partial hepatectomy, and 0.3%, 11%, 33%, 8% and 26% of patients underwent transplantation, ablation, TACE, targeted therapy and best supportive care, respectively.

### Distribution of nomogram points and primary anti-cancer treatments

**Fig. 1:**
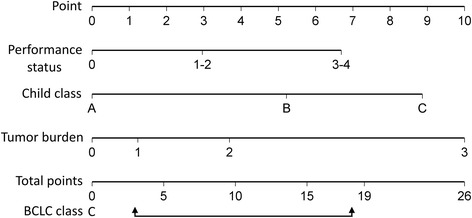
The nomogram is used by adding up the points identified on the scales of these three parameters. BCLC stage C HCC patients have nomogram points between 3 to 18.2

**Fig. 2:**
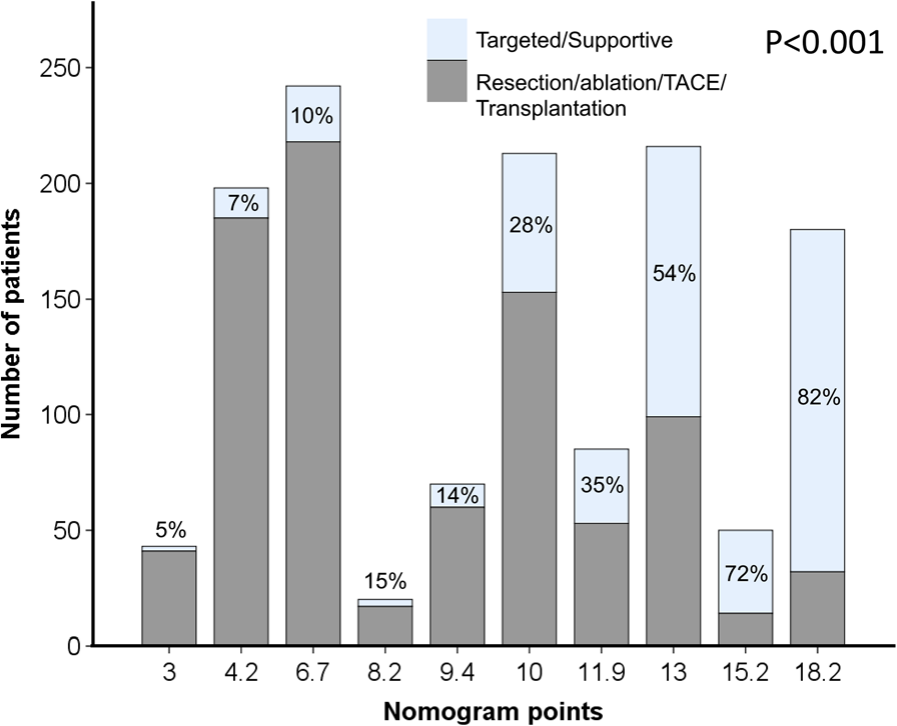
Distribution of nomogram points and percentages of patients receiving aggressive and targeted/supportive treatments. Patients with higher nomogram points are more likely to receive targeted/supportive therapies (*p* < 0.001)

As shown in Fig. 1, nomogram points were assigned to all enrolled patients by using PS, severity of cirrhosis and tumor burden according to our recent study [10]. The range of total nomogram points was between 3 (PS 1–2, CTP class A and tumor burden 0) to 18.2 (PS 1–2, CTP class B and tumor burden 3) for stage C patients (Fig. 2). Ninety-five percent of patients with 3 points underwent aggressive treatments, and 82% of patients with 18.2 points received targeted/supportive therapies. Patients with more advanced HCC had a significantly higher tendency to receive targeted/supportive treatments (*p* <  0.001).

### Characteristics of patients divided by nomogram points

Patients were divided by nomogram points into four groups (total points: 3–4.2, 6.7–10, 11.9–13, 15.2–18.2). Comparisons of baseline characteristics between patients receiving aggressive and targeted/supportive treatments within these four groups are given in Table 2. Within each group, patients receiving different treatments had similar distributions of age, gender, etiology of chronic liver disease and prevalence of diabetes mellitus (all *p* > 0.05), but significant differences in tumor burden, PS, and severity of cirrhosis were found among patients undergoing different forms of treatment within these four groups (*p* < 0.05).

**Table 2:** Comparison of demographics of patients receiving different treatments stratified by nomogram points

Nomogram points	3–4.2 (*n* = 241)			6.7–10 (*n* = 545)			11.9–13 (*n* = 301)			15.2–18 (*n* = 230)		
	SR/Ablation/ TACE/ Transplant (*n* = 226)	Targeted/ Supportive (*n* = 15)	P	SR/Ablation/ TACE/ Transplant (*n* = 448)	Targeted/ Supportive (*n* = 97)	P	SR/Ablation/ TACE/ Transplant (*n* = 152)	Targeted/ Supportive (*n* = 149)	P	SR/Ablation/ TACE/ Transplant (*n* = 46)	Targeted/ Supportive (*n* = 184)	p
Age	67 ± 12	72 ± 10	0.173	65 ± 13	64 ± 15	0.878	64 ± 14	64 ± 15	0.296	59 ± 13	62 ± 13	0.113
Male (%)	166 (73)	14 (93)	0.124	340 (76)	71 (73)	0.576	117 (77)	125 (84)	0.131	36 (78)	162 (88)	0.086
HBV (%)	110 (49)	4 (27)	0.115	238 (53)	55 (57)	0.522	85 (56)	87 (58)	0.665	30 (65)	112 (61)	0.587
HCV (%)	81 (36)	7 (47)	0.399	130 (29)	24 (25)	0.397	27 (18)	33 (22)	0.341	10 (22)	37 (20)	0.806
Alcoholism (%)	47 (21)	2 (13)	0.742	99 (22)	14 (14)	0.091	41 (27)	36 (24)	0.576	12 (26)	57 (31)	0.517
CTP class A (%)	225 (100)	14 (93)	0.121	371 (83)	83 (86)	0.51	99 (65)	117 (79)	0.01	0	0	N/A
Ascites (%)	21 (9)	2 (13)	0.642	83 (19)	20 (21)	0.633	61 (40)	48 (32)	0.153	30 (65)	133 (72)	0.346
eGFR > 60 (ml/min/1.73m^2^)	173 (77)	5 (33)	0.001	338 (75)	71 (73)	0.642	114 (75)	111 (75)	0.92	37 (80)	144 (78)	0.747
Tumor > 5 cm	26 (12)	6 (40)	0.007	280 (63)	75 (77)	0.006	121 (80)	135 (91)	0.008	34 (74)	160 (87)	0.029
Single tumor (%)	167 (74)	8 (53)	0.084	278 (62)	53 (55)	0.175	73 (48)	81 (54)	0.272	23 (50)	89 (48)	0.843
Tumor burden 0/1/2/3	16/69/14/0	13/40/47/0	0.008	4/12/50/34	3/8/27/62	<.001	0/0/35/65	0/0/21/79	0.01	0/0/0/100	0/0/0/100	N/A
Performance status 0/1/2 (%)	17/64/19	47/33/20	0.014	35/47/18	66/21/13	<.001	0/68/32	0/69/31	0.799	30/46/24	20/48/32	0.239
Vascular invasion (%)	0	0	N/A	136 (30)	57 (59)	<.001	84 (55)	107 (72)	0.003	37 (80)	170 (92)	0.016
Metastasis/lymph node (%)	0	0	N/A	27 (6)	15 (15)	0.002	30 (20)	47 (32)	0.019	14 (30)	65 (35)	0.532
AFP > 400 ng/mL	33 (15)	3 (20)	0.475	137 (31)	50 (52)	<.001	68 (45)	92 (62)	0.003	22 (48)	115 (63)	0.07
Diabetes mellitus (%)	76 (34)	6 (40)	0.614	112 (25)	23 (24)	0.79	47 (31)	46 (31)	0.993	10 (22)	38 (21)	0.871

*SR* surgical resection, *TACE* transarterial chemoembolization, *CTP* Child-Turcotte-Pugh, *eGFR* estimated glomerular filtration rate, *HBV* hepatitis B virus, *HCV* hepatitis C virus

### Comparison of survival between patients receiving different treatments stratified by nomogram points

**Fig. 3:**
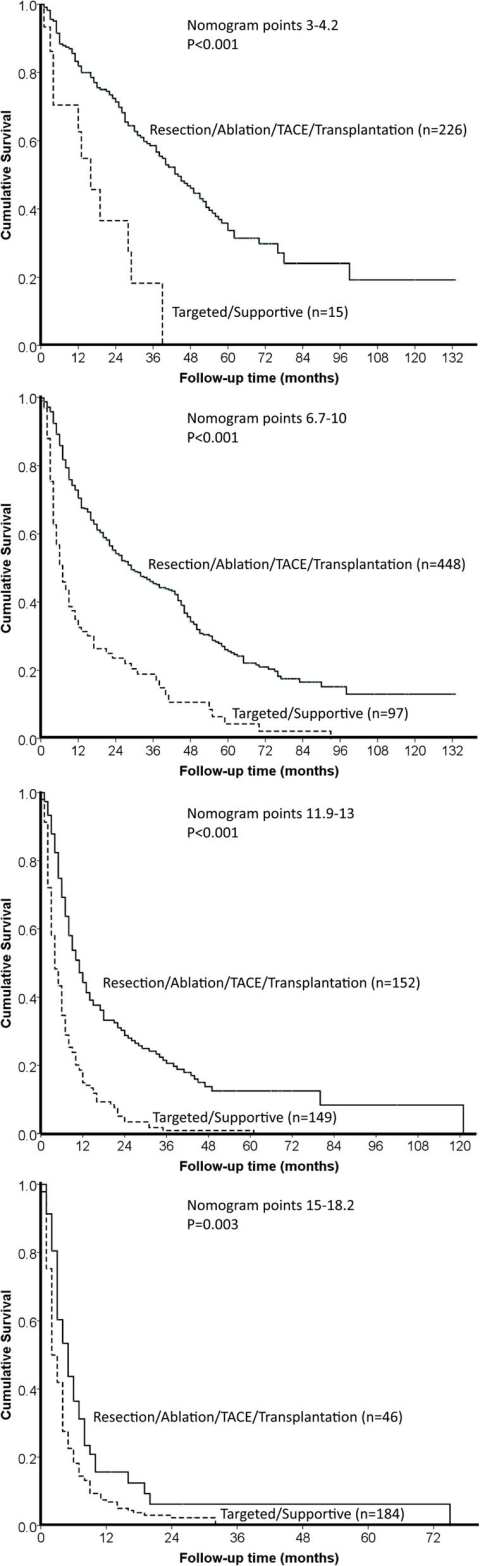
Patients receiving aggressive treatments had significantly better overall survival compared to patients receiving targeted/supportive treatments across four groups divided by nomogram points (all *p* < 0.05)

Within all four groups, patients receiving aggressive treatments had significantly better overall survival compared to patients receiving targeted/supportive therapies (all *p* < 0.05; Fig. 3).

### Characteristics of patients in the propensity model divided by nomogram points

By using the propensity model, 15, 87, 112 and 45 pairs of matched HCC patients were selected for the four groups with absolute differences in propensity score of matched pairs of < 0.0001, 0.0007, 0.0007 and < 0.0001, respectively (Table 3). Among matched patients stratified by the nomogram points, there were no significant differences in gender, etiology of chronic liver disease, severity of cirrhosis, number and size of tumor, PS, and prevalence of diabetes mellitus (all *p* > 0.05). The majority of standardized differences of significant prognostic variables (e.g., tumor burden, cirrhosis, and PS) were controlled under 0.2; some standardized differences in group 1 (nomogram points: 3–4.2) and 4 (nomogram points 15.2–18.2) were larger than 0.2.

**Table 3:** Comparison of demographics of patients receiving different treatments stratified by nomogram points in the propensity model

Nomogram points/Absolute difference in propensity score of matched pairs	3–4.2 (*n* = 30)/< 0.0001			6.7–10 (*n* = 174)/0.0007			11.9–13 (*n* = 224)/0.0007			15.2–18 (*n* = 90)/< 0.0001		
	SR/Ablation/ TACE/ Transplant (*n* = 15)	Targeted/ Supportive (*n* = 15)	P/Stddiff	SR/Ablation/ TACE/ Transplant (*n* = 87)	Targeted/ Supportive (n = 87)	P/Stddiff	SR/Ablation/ TACE/ Transplant (*n* = 112)	Targeted/ Supportive (*n* = 112)	P/Stddiff	SR/Ablation/ TACE/ Transplant (*n* = 45)	Targeted/ Supportive (*n* = 45)	P/Stddiff
Age	75 ± 13	72 ± 10	0.92/0.14	63 ± 14	65 ± 14	0.21/0.18	63 ± 15	64 ± 14	0.06/0.3	59 ± 14	61 ± 13	0.094/0.16
Male (%)	13 (87)	14 (93)	1/0.14	65 (75)	66 (76)	0.86/0.18	84 (75)	93 (83)	0.14/0.3	35 (78)	39 (87)	0.27/0.61
HBV (%)	6 (40)	4 (27)	0.7/0.29	47 (54)	48 (55)	0.88/0.02	64 (57)	65 (58)	0.89/0.02	30 (67)	21 (47)	0.056/0.41
HCV (%)	5 (33)	7 (47)	0.46/0.27	27 (31)	22 (25)	0.4/0.13	19 (17)	23 (21)	0.49/0.09	9 (20)	13 (29)	0.327/0.21
Alcoholism (%)	2 (13)	2 (13)	1/0	10 (11)	13 (15)	0.5/0.1	28 (25)	32 (29)	0.55/0.08	11 (24)	14 (31)	0.48/0.15
CTP class A (%)	15 (100)	14 (93)	1/0.38	72 (83)	74 (85)	0.68/0.06	85 (76)	80 (81)	0.45/0.1	0	0	N/A
Ascites (%)	2 (13)	2 (13)	1/0	15 (17)	18 (21)	0.56/0.09	37 (33)	41 (37)	0.58/0.08	29 (64)	34 (76)	0.25/0.24
eGFR > 60 (ml/min/1.73m^2^)	5 (33)	5 (33)	1/0	62 (71)	65 (75)	0.61/0.08	83 (74)	79 (71)	0.55/0.08	37 (82)	33 (73)	0.311/0.22
Tumor > 5 cm	6 (40)	6 (40)	1/0	66 (76)	65 (75)	0.86/0.03	96 (86)	99 (88)	0.55/0.08	34 (76)	34 (76)	1/0
Single tumor (%)	11 (73)	8 (53)	0.26/0.42	56 (64)	47 (54)	0.17/0.21	57 (51)	50 (45)	0.35/0.13	23 (51)	21 (47)	0.673/0.09
Tumor burden 0/1/2/3	7/53/40/0	13/40/47/0	0.67/0.31	2/13/28/57	3/9/29/59	0.87/0.13	0/0/24/76	0/0/29/71	0.45/0.13	0/0/0/100	0/0/0/100	N/A
Performance status 0/1/2 (%)	40/40/20	47/33/20	0.9/0.15	60/22/18	62/23/15	0.83/0.09	0/71/29	0/71/29	1/0	29/47/24	20/40/40	0.266/0.35
Vascular invasion (%)	0	0	N/A	47 (54)	49 (56)	0.76/0.05	72 (64)	71 (63)	0.89/0.02	37 (82)	37 (82)	1/0
Metastasis/lymph node (%)	0	0	N/A	8 (9)	9 (10)	0.8/0.04	26 (23)	31 (28)	0.44/0.1	13 (29)	21 (47)	0.08/0.37
AFP > 400 ng/mL	3 (20)	3 (20)	1/0	44 (51)	42 (48)	0.76/0.05	65 (58)	61 (54)	0.59/0.07	22 (49)	28 (62)	0.203/0.27
Diabetes mellitus (%)	7 (47)	6 (40)	0.71/0.13	18 (21)	20 (23)	0.71/0.06	32 (29)	39 (35)	0.31/0.13	9 (20)	9 (20)	1/0

*SR* surgical resection, *TACE* transarterial chemoembolization, *Stddiff* standardized difference, *CTP* Child-Turcotte-Pugh, *eGFR* estimated glomerular filtration rate, *HBV* hepatitis B virus, *HCV* hepatitis C virus

### Comparison of survival between patients receiving different treatments stratified by nomogram points in the propensity model

**Fig. 4:**
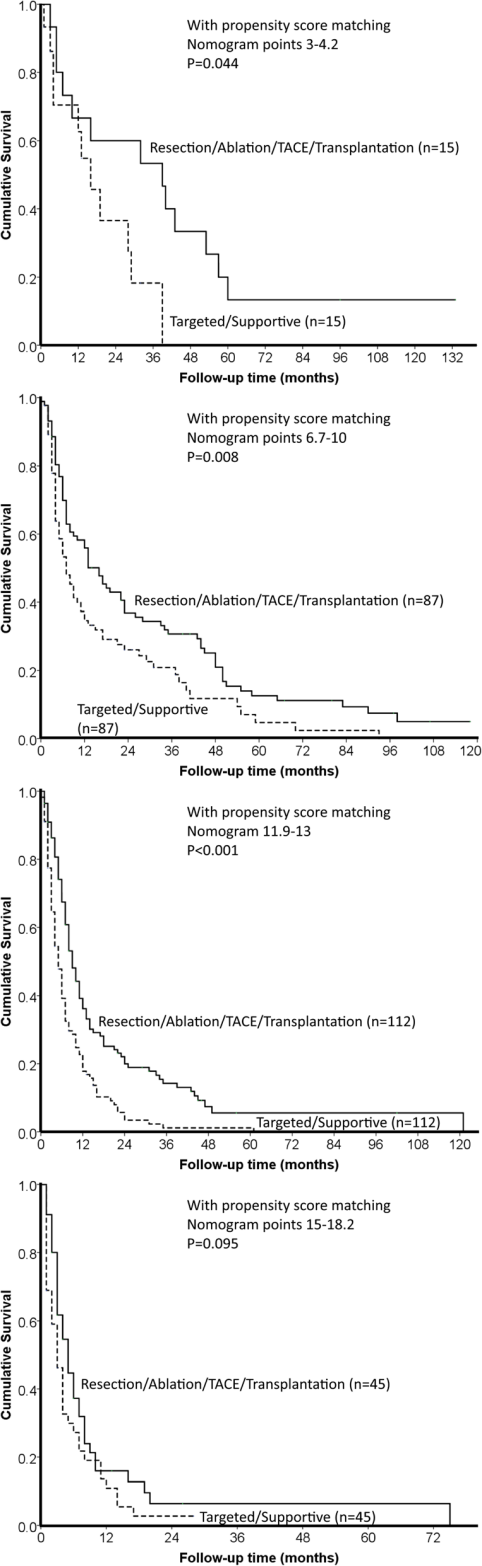
With the propensity score matching model, patients receiving aggressive treatments had significantly better survival when nomogram points were less than 15 (*p* < 0.05). For patients with nomogram more than 15, there was no significant difference in survival between patients receiving aggressive and targeted/supportive therapies (*p* > 0.05)

For patients with nomogram points 3–4.2, 6.7–10 and 11.9–13, aggressive treatments were associated with significantly better survival (all *p* < 0.05). For patients with nomogram points 15.2–18, there were no significant differences in long-term survival after all variables were controlled by the propensity model (*p* > 0.05; Fig. 4).

## Discussion

Patients with advanced (BCLC stage C) HCC usually have remarkably complex compositions. As such, individualized treatment strategy by a multidisciplinary team has been encouraged. The benefits of aggressive treatments for patients with advanced HCC have been reported by quite a few studies [18, 19]. In this study, we investigated a large patient cohort to provide objective and quantitative recommendations to help patients and physicians to decide if aggressive treatments might be helpful to prolong overall survival. By applying our recently proposed BCLC nomogram, we have disclosed an easy-to-use cutoff to identify patients with BCLC stage C who can potentially benefit from aggressive treatments. This finding also has important clinical implications because our results may help avoid unnecessary treatment associated severe complications.

The BCLC staging system is mainly determined by tumor burden, severity of cirrhosis and PS. For BCLC stage C, PS 1–2 and tumor burden 3 (lymph node involvement, vascular invasion, or distant metastasis) are sufficient criteria, and CTP class C cirrhosis is only the exclusion criterion regarding the severity of cirrhosis. Therefore, the design of BCLC system results in a very heterogeneous composition of BCLC stage C patients [6]. For example, a patient with a single small nodule, minimal cirrhosis and PS 1 is classified as BCLC stage C, the same cancer stage as a patient with large tumors, vascular invasion, CTP class B cirrhosis and PS 2; these patients generally have highly variable clinical course and prognosis. The nomogram of BCLC assigned points from 3 to 18 to patients with BCLC stage C and predicted their 3-year survival rate from approximately 80% to 10%, respectively [10]. These findings underscore how diverse this patient population is, and justify the necessity of treatment planning on an individual basis. The percentages of aggressive and targeted/supportive treatments (Fig. 2) in our study clearly showed that patients with smaller tumor burden, less severe cirrhosis, and better physical condition were more likely to undergo aggressive therapies, indicating individualized treatment strategy has been applied in the daily practice.

We further split study patients into four groups (approximately one-quarter in each) by the nomogram points to specifically investigate the prognostic effect of aggressive treatments. In these four groups, patients receiving different therapies (aggressive vs. targeted/supportive) had similar baseline characteristics including age, gender, etiology of chronic liver disease and prevalence of diabetes mellitus at enrollment. On the other hand, several significant differences of treatment allocation-related variables (including severity of cirrhosis, tumor burden, and PS) were found between patients receiving different treatments among the four groups, suggesting therapeutic strategy is highly variable even when patients have very similar nomogram points.

Notably, patients undergoing aggressive treatments had significantly better outcome compared to patients undergoing targeted/supportive treatments in this study; however, patients who received aggressive treatments more often had favorable baseline characteristics and discreetly selected by the multidisciplinary team. Therefore, we calculated propensity scores and performed one-to-one match to minimize the confounding effects of baseline differences, and introduced matched pairs of patients into survival analyses again. With the propensity model, matched pairs of patients were selected from the four groups, and all the survival-related variables were comparable. Survival analyses using paired matches of patients showed that patients with nomogram points less than 15 could benefit from aggressive treatments. Alternatively, for patients with more advanced BCLC stage C HCC (nomogram points > 15), there was no significant difference in survival between aggressive and targeted/supportive treatment groups.

For BCLC stage C patients with relatively favorable characteristics (PS 1, CTP class A or no cirrhosis, and smaller tumor burden), patients and physicians tend to choose more aggressive forms of treatment. Surgical resection is reported to prolong survival for patients with advanced yet resectable HCCs [5, 20], and ablation has been administered in selected patients beyond the Milan criteria [21]. Moreover, TACE has been associated with improvement of overall survival compared to targeted therapy [18]. All these studies support the idea of more aggressive managements for patients with advanced HCC [22, 23]. Also, our previous studies showed that patients with PS 1 and 2 could have increased survival after aggressive treatments [6, 24]. Altogether, targeted therapy might not be the optimal treatment choice for all BCLC stage C HCC patients. Importantly, our findings from the propensity model not only support the benefits of individualized treatments but also provide an easy-to-use cutoff nomogram point to help patients on treatment selection when taking potential complications of aggressive treatments into consideration.

This study has some limitations. Firstly, aggressive treatments could have differential survival impact to some extent. Similarly, patients receiving targeted therapy or supportive treatment may have different survival distribution, and further studies are required to clarify this issue [25, 26]. Secondly, only four patients in this study received liver transplantation. Therefore the prognostic effect of transplantation for BCLC stage C patients might not be accurately evaluated [27, 28]. Thirdly, multiple studies recently published promising results regarding the use of immunotherapy to prolong overall survival of HCC patients; the application of immunotherapy is not addressed in this study [29, 30]. Lastly, some patients underwent multiple sessions of anti-cancer treatment, but its effect was not taken into account in this study. The prognostic effect of follow-up treatment warrants future investigations.

## Conclusions

In conclusion, this study indicates that the nomogram of BCLC could provide useful stratification for patients with advanced HCC. By applying the nomogram in our large patient cohort, the distribution of individualized treatment based on disease severity can be clearly specified. With the propensity score matching model, we propose an easy-to-use cutoff nomogram point to facilitate treatment decision, avoid unnecessary medical interventions, and improve overall survival in patients with advanced HCC.

## Abbreviations

AFP: α-fetoprotein
BCLC: Barcelona Clinic Liver Cancer
CTP: Child-Turcotte-Pugh
eGFR: estimated glomerular filtration rate
HBV: Hepatitis B virus
HCC: Hepatocellular carcinoma
HCV: Hepatitis C virus
PS: Performance status
RFA: Radiofrequency ablation
TACE: Transarterial chemoembolization
